# Spatio-Temporal Characterization of Cellular Senescence Hallmarks in Experimental Ischemic Stroke

**DOI:** 10.3390/ijms26052364

**Published:** 2025-03-06

**Authors:** Júlia Baixauli-Martín, Maria Consuelo Burguete, Mikahela A. López-Morales, María Castelló-Ruiz, Alicia Aliena-Valero, Teresa Jover-Mengual, Dianoush Falahatgaroshibi, Germán Torregrosa, Juan B. Salom

**Affiliations:** 1 Unidad Mixta de Investigación Cerebrovascular, Instituto de Investigación Sanitaria La Fe, Hospital Universitario y Politécnico La Fe, 46026 Valencia, Spain; juliabaixauli@gmail.com (J.B.-M.); m.consuelo.burguete@uv.es (M.C.B.); maria.castello@uv.es (M.C.-R.); alicia_aliena@iislafe.es (A.A.-V.); teresa.jover@uv.es (T.J.-M.); dianosh.felahatgar11@gmail.com (D.F.); gtorregrosab@gmail.com (G.T.); salom_jba@gva.es (J.B.S.); 2 Departamento de Fisiología, Universidad de Valencia, 46100 Burjassot, Spain; 3 Departamento de Fisioterapia, Universidad de Valencia, 46010 Valencia, Spain; 4 Departamento de Biología Celular, Biología Funcional y Antropología Física, Universidad de Valencia, 46100 Burjassot, Spain; 5 The Saul R. Korey Department of Neurology and Dominick P. Purpura Department of Neuroscience, Albert Einstein College of Medicine, New York, NY 10461, USA; 6 Departamento de Biotecnología, Universidad Politécnica de Valencia, 46022 Valencia, Spain

**Keywords:** cell cycle arrest, cellular senescence, DNA damage, ischemic stroke, middle cerebral artery occlusion, senescence-associated β-galactosidase, senescence-associated secretory phenotype

## Abstract

In recent years, evidence of the existence of cellular senescence in the central nervous system has accumulated. In ischemic stroke, cellular senescence has been suggested as an unidentified pathophysiological mechanism, prompting research into the neuroprotective potential of senolytic drugs. This study aims to provide spatio-temporal evidence of the existence of brain senescence following ischemic stroke and to elucidate the involved pathways and cell types. We focused on the most established markers of senescence: cell cycle arrest (p16, p21); lysosomal activity (senescence-associated β-galactosidase [SA-β-gal]); the senescence-associated secretory phenotype ([SASP]; Interleukin-6 [IL-6], Interleukin-1β [IL-1β], Tumor necrosis factor [TNF]); and DNA/nuclear damage (Checkpoint kinase 1 [Chk1], Checkpoint kinase 2 [Chk2], Lamin B1 [LB1]). Male Wistar rats underwent 60 min of transient middle cerebral artery occlusion, followed by 24 h and 3, 7, and 14 days of recovery. Our results show significant increases in p16 expression, particularly in neurons and microglia/macrophages; SA-β-gal accumulation in the infarcted tissue; significant increases in SASP markers as early as 24 h after reperfusion; and significant changes in Chk1, Chk2, and LB1 at 14 days. Overall, our findings lend support to the existence of senescence after ischemic stroke in neurons and microglia/macrophages. However, there is still room to gain further insight into the role of senescence in the pathophysiology of ischemic stroke and in the implementation of successful senolytic therapy.

## 1. Introduction

With ~12 million new cases/year, ~100 million survivors, 6.5 million deaths/year, and 143 million years of healthy life lost, stroke remains a major disease worldwide [1]. The highest percentage of these figures corresponds to ischemic stroke. The standard therapeutic approach for ischemic stroke is the removal of the clot via intravenous thrombolysis with a recombinant tissue plasminogen activator, via mechanical endovascular thrombectomy, or a combination of both. Although some inadequacies limit the widespread use of these treatments, placing a catheter in a reopened cerebral artery offers the opportunity to apply neuroprotective treatments exactly when and where they may exert their optimal benefits [2,3,4]. As recently emphasized by Eren and Yilmaz, an effective neuroprotective strategy can not only prolong the indication period of recanalization treatments in the acute stage of ischemic stroke but also reduce neuronal necrosis and protect the brain against reperfusion injury [5]. Although none of the neuroprotectors tested so far have been promoted or approved, finding and developing new targets for neuroprotective intervention is still the focus and goal of research in this field.

First proposed to describe the limited replicative ability of human fibroblasts, the term “cellular senescence” now describes a functional state in which the cell acquires a particular phenotype, which has a double face: on the one hand, it constitutes a homeostatic mechanism in important physiological processes (elimination of damaged cells, tissue regeneration, participation in development, etc.); on the other hand, there is evidence of its contribution to the functional deterioration associated with aging, as well as the development of related diseases [6]. The general hallmarks of cellular senescence have been described [7] and include (1) stable cell cycle arrest; (2) increased lysosomal activity (overexpression of senescence-associated β-galactosidase [SA-β-gal]); (3) strong paracrine secretion (senescence-associated secretory phenotype [SASP]); (4) macromolecular damage and metabolic adaptations (dysfunctional mitochondria, increased reactive oxygen species [ROS]); (5) DNA/nuclear damage (DNA damage response [DDR] activation, chromatin reorganization, changes in the nuclear envelope); and (6) morphological changes (enlarged and flattened cell shape).

In recent years, evidence has been accumulating that confirms the existence of cellular senescence in the central nervous system, mainly in the context of neurodegenerative diseases such as Parkinson’s and Alzheimer’s diseases [8,9]. The cell types whose senescence has been revealed are neurons, astrocytes, and microglia [10]. In the last few years, we and others have reported evidence that cellular senescence could be a hitherto unidentified pathophysiological mechanism in ischemic stroke [11,12,13,14]. Therefore, a new line of research has been opened on the neuroprotective potential of drugs able to eliminate senescent cells [15,16,17,18].

The aim of the present study is twofold: firstly, to provide spatio-temporal evidence of the existence of cellular senescence following ischemic stroke and, secondly, to elucidate the pathways and cell types involved in the development of brain senescence after ischemic stroke. In order to achieve those objectives, we have focused on (1) the expression of the cell cycle arrest markers p16 and p21; (2) lysosomal activity through SA-β-gal staining; (3) the expression of SASP markers (Interleukin-6 [IL-6], Interleukin-1 β [IL-1β], and Tumor necrosis factor [TNF]); and (4) the expression of DDR markers (Checkpoint kinases 1 [Chk1] and 2 [Chk2]) and the nuclear envelope component Lamin B1 (LB1).

We have used the rat filament model of transient middle cerebral artery occlusion (tMCAO) as the animal version of the thrombectomy technique used to treat large vessel occlusion in stroke patients. Brain regions differentially affected by the ischemic insult were analyzed (non-ischemic, infarcted, and peri-infarcted tissue). Specific brain cell types expressing the markers were identified (neurons, microglia/macrophages, and astrocytes). Finally, the development of senescence markers at time-points from 24 h to 14 days after the ischemic stroke was assessed.

## 2. Results

All animals included in the study exhibited comparable physiological conditions during the three stages of tMCAO (baseline, ischemia, and reperfusion) and demonstrated similar ischemic brain damage, as evidenced by their neurofunctional scores, in line with similar ischemia–reperfusion (I/R) patterns (Table S1).

### 2.1. Cell Cycle Arrest: p16 and p21 Pathways

**Figure 1:**
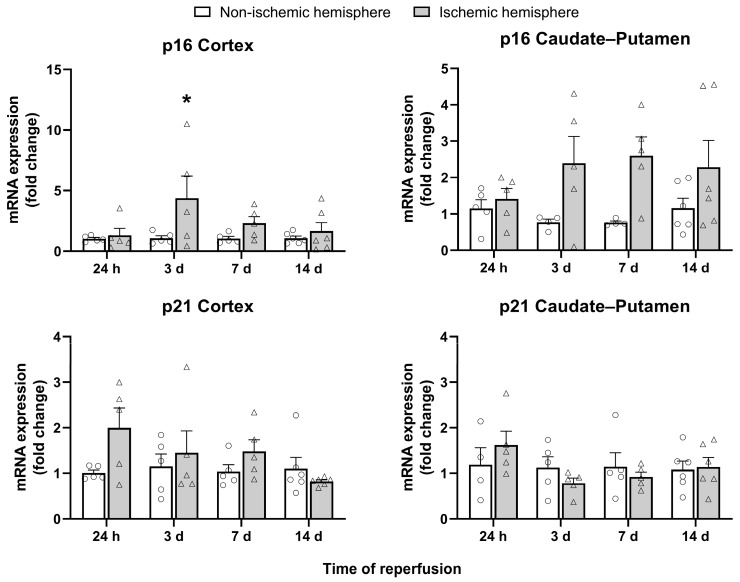
mRNA expression of the cell cycle arrest markers p16 and p21 in the non-ischemic (circles) and ischemic (triangles) cortex and caudate–putamen samples at 24 h and 3, 7, and 14 days post-I/R rats. The results are expressed as fold change and represented as mean ± standard error of the mean (SEM) from *n* = 5 (24 h), *n* = 5 (3 days), *n* = 5 (7 days), and *n* = 6 (14 days) animals. Statistical analysis: two-way ANOVA followed by Sidák multiple comparison test. * *p* < 0.05, significantly different from the corresponding non-ischemic hemisphere.

Cell cycle arrest is a major hallmark of cellular senescence; gene expression of p16 and p21 was assessed at different I/R times: 24 h and 3, 7, and 14 days (Figure 1). With regard to p16, it was significantly overexpressed at 3 days post-I/R in the cortex of the ischemic hemisphere (*p* < 0.05) and gradually decreased until the last I/R time analyzed. In the caudate–putamen, a tendency toward their overexpression was observed at 3 days post-I/R, and these levels remained elevated and stable until 14 days post-I/R. Regarding p21, no significant changes were observed in its expression at the four I/R times in either the cortex or caudate–putamen.

**Figure 2:**
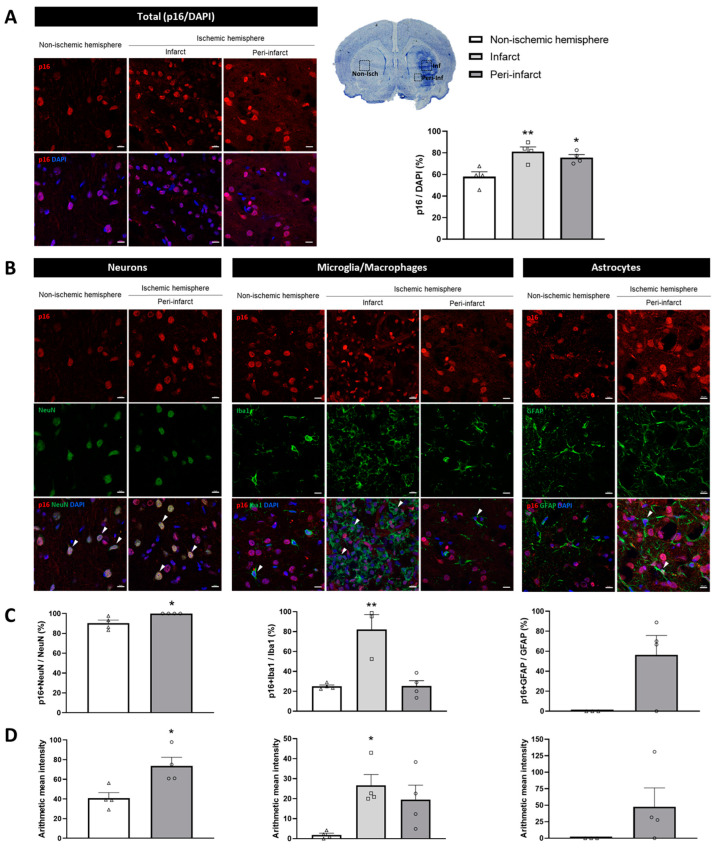
The expression of the cell cycle marker p16 in brain samples from 14-day post-I/R rats. (**A**) Representative immunofluorescent images (**left**) and quantification (**right**) of p16-immunopositive cells with respect to DAPI in the ischemic (infarct [squares] and peri-infarct [circles] regions) and non-ischemic (triangles) hemispheres. (**B**) Immunofluorescent colocalization (arrows) of p16 (red) with the different brain cells (green)—neurons (NeuN), microglia/macrophages (Iba1), and astrocytes (GFAP)—in the three studied regions. (**C**) Quantification of p16-immunopositive neurons, microglia/macrophages, and astrocytes. (**D**) The fluorescence intensity of p16 labeling for each of the specific cell types. Data are expressed as the mean ± standard error of the mean (SEM) from *n* = 4 animals. Statistical analysis: one-way ANOVA followed by Dunnet’s post hoc test (three regions); two-tailed Student’s *t*-test (two regions). Scale bar = 10 μm. * *p* < 0.05; ** *p* < 0.01, significantly different from the non-ischemic hemisphere.

**Figure 3:**
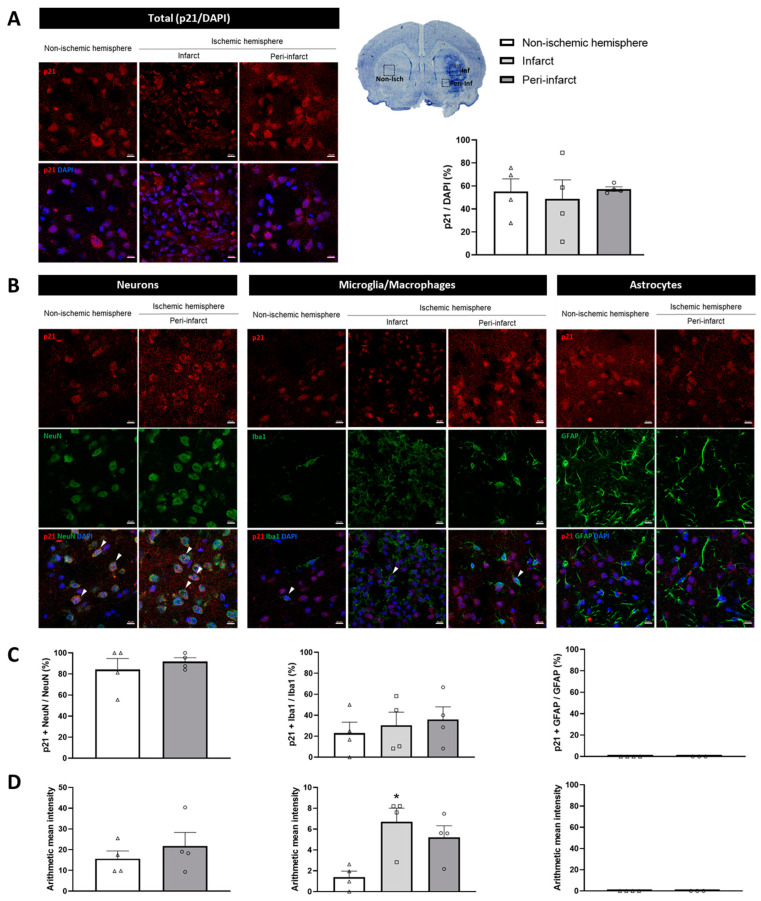
Expression of the cell cycle marker p21 in brain samples from 14-day post-I/R rats. (**A**) Representative immunofluorescent images (**left**) and quantification (**right**) of p21-immunopositive cells with respect to DAPI in the ischemic (infarct [squares] and peri-infarct [circles] regions) and non-ischemic [triangles] hemispheres. (**B**) Immunofluorescent colocalization (arrows) of p21 (red) with the different brain cells (green)—neurons (NeuN), microglia/macrophages (Iba1), and astrocytes (GFAP)—in the three studied regions. (**C**) Quantification of p21-immunopositive neurons, microglia/macrophages, and astrocytes. (**D**) The fluorescence intensity of p21 labeling for each of the specific cell types. Data are expressed as the mean ± standard error of the mean (SEM) from *n* = 4 animals. Statistical analysis: one-way ANOVA followed by Dunnet’s post hoc test (three regions); two-tailed Student’s *t*-test (two regions). Scale bar = 10 μm. * *p* < 0.05, significantly different from the non-ischemic hemisphere.

To gain insight into the expression of p16 and p21 at the protein level with greater spatial resolution in relation to ischemic damage, these markers were also analyzed by immunofluorescence at the longest I/R time (14 days). The number of p16-immunopositive cells significantly increased in the infarct (81.14 ± 8.78%, *p* < 0.01) and peri-infarct (75.69 ± 5.46%, *p* < 0.05) regions of the ischemic hemisphere when compared with the non-ischemic hemisphere (57.97 ± 9.11%; Figure 2A). In contrast, the number of p21-immunopositive cells in the infarct (48.78 ± 32.96%) and peri-infarct (57.32 ± 3.88%) regions did not differ from that in the non-ischemic hemisphere (55.31 ± 21.84%; *p* = 0.8905 and *p* = 0.9888, respectively; Figure 3A). Taken together, these results suggest that cell cycle arrest occurs following ischemic stroke and is mediated by the p16 pathway.

Of particular interest is the expression of the cell cycle inhibitors p16 and p21 on a cell-type basis. So, we then assessed the colocalization of these markers with the main neural cell types: neurons (NeuN-positive), microglia (Iba1-positive), and astrocytes (GFAP-positive).

Firstly, the distribution of the different cell types in the non-ischemic and ischemic hemispheres was examined. As expected, the number of neurons was drastically reduced in the infarct region compared to the non-ischemic hemisphere and the peri-infarct region (Figure S1A). In contrast, the number of microglia/macrophages was significantly higher in the infarct region compared to the other regions studied (Figure S1B). The distribution of astrocytes was similar within the analyzed regions. Nevertheless, this cell type exhibited a clear damaged-like morphology in the infarct region (Figure S1C). In consideration of the aforementioned, the infarct region was excluded from the quantification of senescence markers in neurons and astrocytes.

Secondly, an analysis of the cell cycle inhibitors in the different neural cell types was conducted, revealing that 100.00 ± 0.00% of neurons in the peri-infarct region were p16-immunopositive, compared to 90.19 ± 6.54% in the non-ischemic hemisphere (*p* < 0.05). Regarding p16-immunopositive microglia/macrophages, the proportion observed in the peri-infarct region (25.26 ± 10.91%) was similar to that in the non-ischemic hemisphere (24.97 ± 2.89%; *p* = 0.9994). However, there was a significant increase in the proportion of p16-immunopositive microglia/macrophages within the infarct region, reaching 82.13 ± 25.76% (*p* < 0.01). The presence of p16-immunopositive astrocytes was not identified in the non-ischemic hemisphere. However, in the peri-infarct region, 56.39 ± 38.85% of astrocytes exhibited p16 immunopositivity, although this difference did not reach statistical significance (*p* = 0.0577; Figure 2B,C). The fluorescence intensity of p16 labeling was also assessed in the different cell types. Interestingly, the profile observed in the quantification of p16-immunopositive cells was replicated, with a significant increase in p16 fluorescence intensity noted in neurons within the peri-infarct region (*p* < 0.05) and in microglia/macrophages within the infarct region (*p* < 0.05; Figure 2D).

In relation to the expression of p21 in the three different cell types studied, no changes in the number of either p21-immunopositive neurons or microglia/macrophages were found in the ischemic hemisphere when compared with that in the non-ischemic hemisphere. Finally, p21-immunopositive astrocytes were not identified in either the non-ischemic hemisphere or the ischemic hemisphere (infarct and peri-infarct regions; Figure 3B,C). As to the fluorescence intensity of p21 labeling in the different cell types, a significant increase was observed only in microglia/macrophages in the infarct region (*p* < 0.05; Figure 3D). These findings at the cellular level suggest that neurons and microglia/macrophages undergo cell cycle arrest following ischemic stroke, a process that is distinctive of cell senescence.

### 2.2. Lysosomal Activity: SA-β-Gal Expression

**Figure 4:**
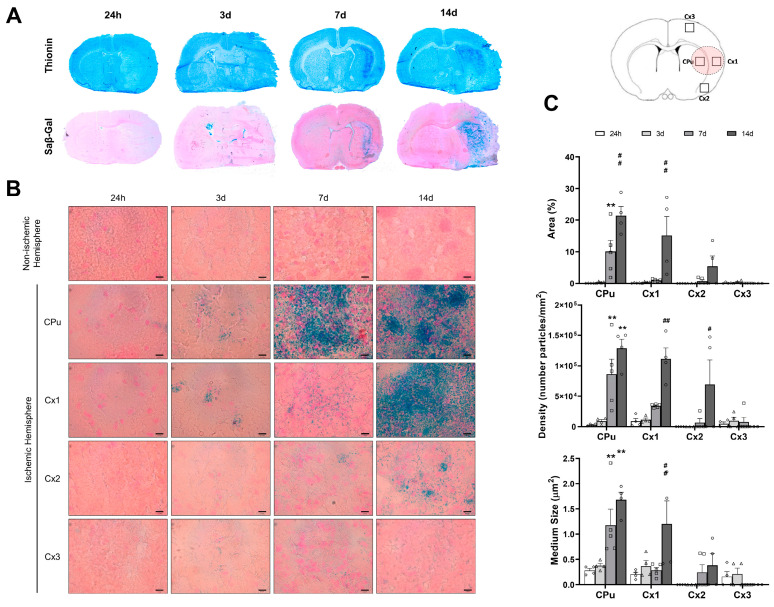
Spatial and temporal distributions of SA-β-gal staining in brain samples from 24-h (diamonds) and 3- (triangles), 7- (squares), and 14-day (circles) post-I/R rats. (**A**) Macroscopic images showing thionin (upper row) and SA-β-gal (lower row) staining of representative coronal brain sections. Note the correspondence between the infarcted and SA-β-gal-positive tissues throughout the entire reperfusion period. (**B**) Microscopic images showing SA-β-gal expression (blue). Four anatomical regions are depicted in the upper-right brain image. The light-pink circle indicates the most representative infarcted area, which generally includes the caudate–putamen (CPu) and parietal cortex (Cx1). Moving away from the infarct, the piriform cortex (Cx2) and frontal cortex (Cx3) are found. (**C**) Quantification of SA-β-gal in terms of relative area, particle density, and particle size. Data are expressed as the mean ± standard error of the mean (SEM) from *n* = 4 (24 h), *n* = 4 (3 days), *n* = 5 (7 days), and *n* = 4 (14 days) animals. Statistical analysis: two-way ANOVA followed by Tukey’s post hoc test. Scale bar = 10 μm. ** *p* < 0.01, significantly different from 3 days post-I/R; ^#^ *p* < 0.05, significantly different from 7 days post-I/R; ^##^ *p* < 0.01, significantly different from 7 days post-I/R.

SA-β-gal expression is a hallmark indicator of the existence of cellular senescence. Macroscopic spatio-temporal inspection of coronal brain slices showed that the expression of SA-β-gal increased in accordance with the number of I/R days, reaching a maximum at 14 days of I/R. SA-β-gal staining was observed to be confined to the infarcted tissue of the ischemic hemisphere, as evidenced by thionin staining (Figure 4A). This was particularly evident in the caudate–putamen and adjacent parietal cortex (Cx1), which are the most affected regions by focal ischemia (Figure 4A).

Microscopic spatio-temporal examination showed no or very scarce SA-β-gal-positive cells at 24 h and 3 days post-I/R in the caudate–putamen, as well as at 24 h, 3 days, and 7 days post-I/R in the infarcted cortex (Cx1). By contrast, massive blue staining of cells was observed both in the caudate–putamen, starting at 7 and reaching a peak at 14 days post-I/R, and in the cortex at 14 days post-I/R (Figure 4B). At 14 days post-I/R, the time with higher SA-β-gal staining, low blue staining was evidenced in the areas remote from the infarct (Cx2 and Cx3). The results of automated image analysis to quantify SA-β-gal particles demonstrated that the total area, density, and size of particles were significantly higher in the caudate–putamen at 7 (*p* < 0.01) and 14 days post-I/R (*p* < 0.01), as well as in Cx1 at 14 days post-I/R (*p* < 0.01), in comparison to the other time-points studied (Figure 4C). These findings, together with those of cell cycle arrest, suggest that cell senescence progressively develops after ischemic stroke in brain regions with ischemic damage.

### 2.3. SASP Markers

**Figure 5:**
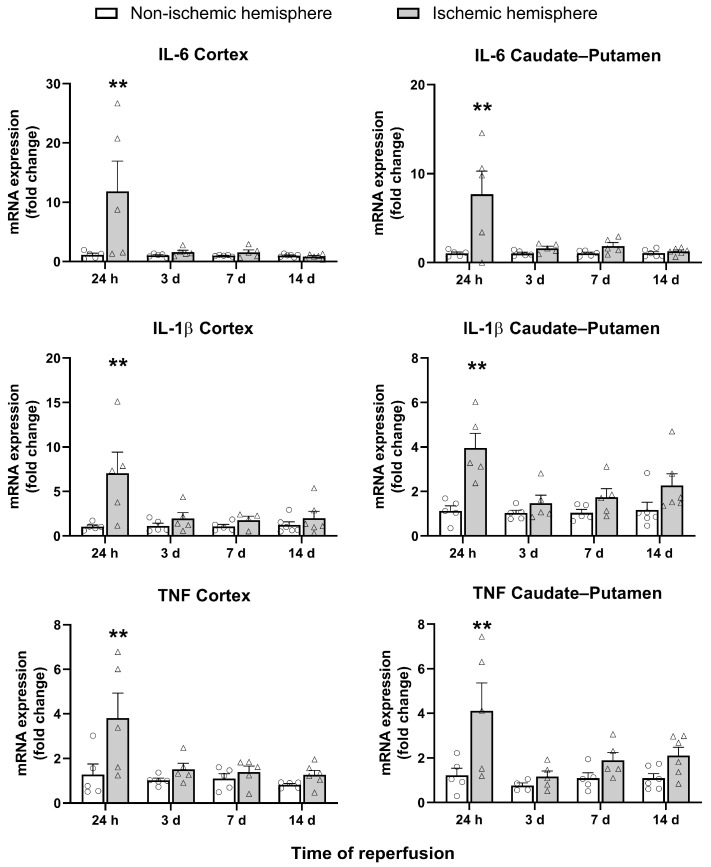
mRNA expression of the SASP markers IL-6, IL-1β, and TNF in non-ischemic (circles) and ischemic (triangles) cortex and caudate–putamen samples from 24-h and 3-, 7-, and 14-day post-I/R rats. The results are expressed as fold change and represented as mean ± standard error of the mean (SEM) from *n* = 5 (24 h), *n* = 5 (3 days), *n* = 5 (7 days), and *n* = 6 (14 days) animals. Statistical analysis: two-way ANOVA followed by Sidák multiple comparison test. ** *p* < 0.01, significantly different from the corresponding non-ischemic hemisphere.

Gene expression of the SASP cytokines IL-6, IL-1β, and TNF was examined at the different post-I/R times. The three cytokines were found to be significantly overexpressed at 24 h post-I/R in both the cortex and caudate–putamen of the ischemic hemisphere when compared to the non-ischemic hemisphere (*p* < 0.01). At 3 days post-I/R, the mRNA levels of cytokines tended to return to baseline levels. Of note, at 7 and 14 days post-I/R, IL-1β and TNF showed a tendency toward a progressive increase in the caudate–putamen, although this was not statistically significant (Figure 5).

### 2.4. DNA and Nuclear Envelope Damage

**Figure 6:**
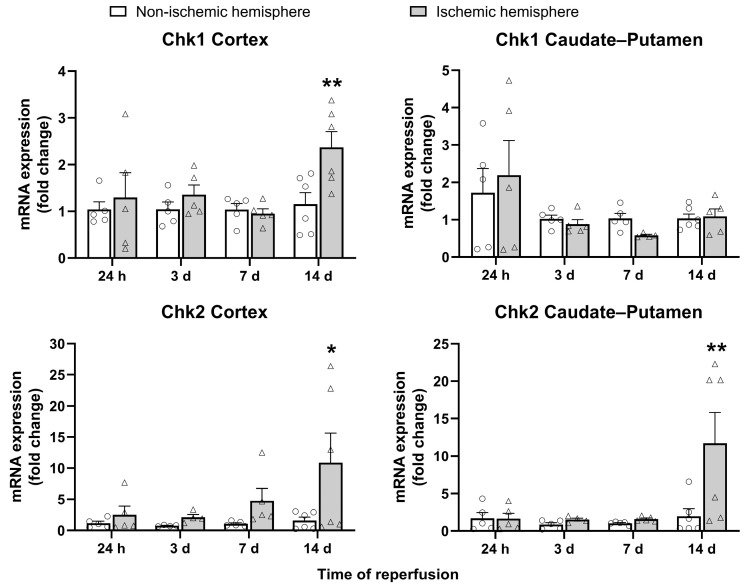
mRNA expression of the DNA damage response markers Chk1 and Chk2 in non-ischemic (circles) and ischemic (triangles) cortex and caudate–putamen samples from 24-h and 3-, 7-, and 14-day post-I/R rats. The results are expressed as fold change and represented as mean ± standard error of the mean (SEM) from *n* = 5 (24 h), *n* = 5 (3 days), *n* = 5 (7 days), and *n* = 6 (14 days) animals. Statistical analysis: two-way ANOVA followed by Sidák multiple comparison test. * *p* < 0.05; ** *p* < 0.01, significantly different from the corresponding non-ischemic hemisphere.

Gene expression of the DDR markers Chk1 and Chk2 was investigated at the different post-I/R times. Significant increases in the expression of both markers were observed at 14 days post-I/R, with Chk1 exhibiting elevated levels in the cortex (*p* < 0.01) and Chk2 exhibiting elevated levels in both the cortex (*p* < 0.05) and the caudate–putamen (*p* < 0.01; Figure 6).

**Figure 7:**
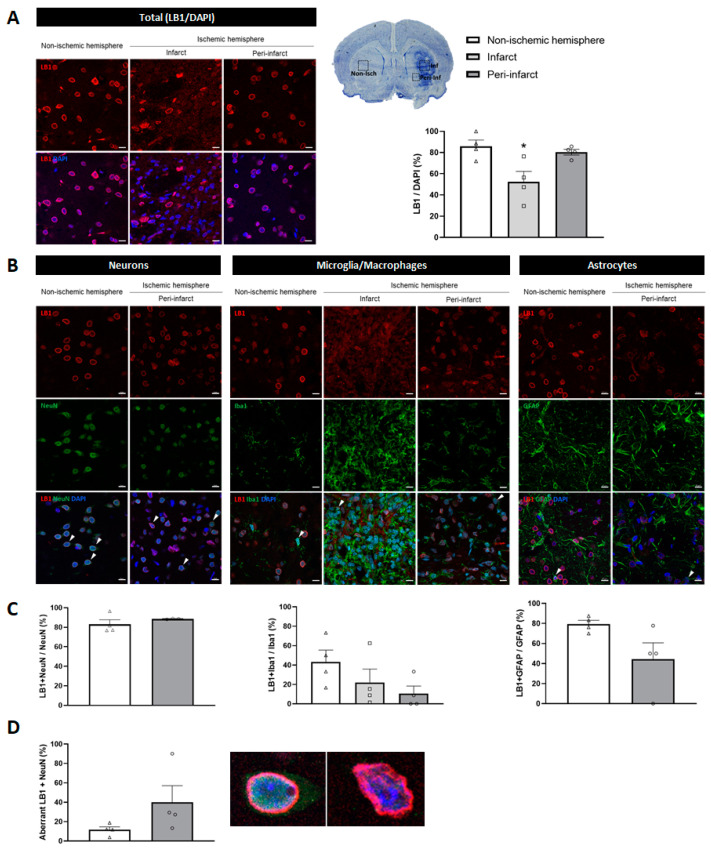
Expression of LB1 in brain samples from 14-day post-I/R rats. (**A**) Representative immunofluorescent images (**left**) and quantification (**right**) of LB1-immunopositive cells with respect to DAPI in the ischemic (infarct [squares] and peri-infarct [circles] regions) and non-ischemic (triangles) hemispheres. (**B**) Immunofluorescent colocalization (arrows) of LB1 (red) with the different brain cells (green): neurons (NeuN), microglia/macrophages (Iba1), and astrocytes (GFAP). (**C**) Quantification of LB1-immunopositive neurons, microglia/macrophages, and astrocytes. (**D**) Representative images and quantification of LB1-immunopositive normal and aberrant neuronal nuclear envelopes. Data are expressed as the mean ± standard error of the mean (SEM) from *n* = 4 animals. Statistical analysis: one-way ANOVA followed by Dunnet’s post hoc test (three regions); two-tailed Student’s *t*-test (two regions). Scale bar = 10 μm. * *p* < 0.05, significantly different from the non-ischemic hemisphere.

The nuclear envelope was also assessed by immunofluorescence detection of LB1 at 14 days post-I/R. The number of LB1-immunopositive nuclei significantly dropped in the infarct region of the ischemic hemisphere compared to the non-ischemic one (52.50 ± 19.47% versus 85.92 ± 11.67%; *p* < 0.05). No changes were found in the number of LB1-immunopositive nuclei in the peri-infarct region (80.38 ± 5.52%; *p* = 0.7903; Figure 7A). Regarding the expression of LB1 in the neural cell types, no changes in the number of LB1-immunopositive neurons were observed between the non-ischemic and ischemic hemispheres. However, in microglia/macrophages and astrocytes, a decreasing trend was observed in the ischemic hemisphere (Figure 7B,C). The morphology of the LB1-immunopositive nuclei was also assessed in the different cell types, and no changes in LB1 morphology were observed in either microglia/macrophages or astrocytes. Nevertheless, the nuclei of some neurons in the peri-infarct region exhibited a folded and wavy structure, which we considered an aberrant nuclear envelope, in contrast to the spherical shape usually observed in the non-ischemic hemisphere (Figure 7D). Although the number of aberrant neuronal nuclei was different (11.61 ± 3.05% versus 40.00 ± 17.04% in non-ischemic and peri-infarct regions, respectively), the increase did not reach statistical significance (*p* = 0.1521; Figure 7D).

## 3. Discussion

Cellular senescence has been proposed to be a pathophysiological event after ischemic stroke. The identification and establishment of universal markers of the senescent phenotype in vivo represents a significant challenge due to the high variability observed in this process. It has recently been proposed that the identification of a minimum of three senescence markers representing different properties of this process would confirm the existence of cellular senescence, as long as at least one of those markers indicates cell cycle arrest promoted by the p16 or p21 pathway [19]. In the present study, we have spatio-temporally characterized the expression of the most commonly used markers of cellular senescence after an episode of ischemic stroke and found the following: (1) significant increases in the expression of the cell cycle arrest marker p16; (2) clear-cut identification of SA-β-gal accumulating in the infarcted brain tissue; (3) significant increases in the expression of the SASP markers IL-6, IL-1β, and TNF; (4) significant increases in the expression of Chk1 and Chk2; and (5) a significant reduction in LB1 levels.

Cell cycle arrest is a major hallmark of cellular senescence. Our results show that after an ischemic stroke, an increase in p16 but not in p21 gene expression occurs at 3 days post-I/R. Increased expression of p16 has also been reported in rats at 4 days post-ischemia [16] and in mice at 24 h [15,17] or 3 days [12] post-ischemia. The same has been evidenced for the p21 inhibitor at 3 days post-ischemia [12], which does not correspond with our results. Since the increased p16 expression persists for up to 14 days post-I/R at the protein level, our results point to the involvement of p16 instead of that of p21 in the development of brain cell senescence after stroke. This is consistent with previous reports showing that stress-induced premature senescence and its maintenance develop through p16, in contrast to the p21 pathway, which appears to have an initiating role [20,21].

SA-β-gal activity is one of the most widely used biomarkers of cellular senescence [22]. Our results show increasing blue staining, indicative of SA-β-gal activity, from 7 to 14 days post-I/R in both caudate–putamen and cortex, overlapping with the infarcted tissue. However, the reaction manifested earlier and with greater intensity in the caudate–putamen than in the cortex. These results are consistent with those previously reported in a traumatic brain injury model wherein a comparable temporal profile to ours was studied, and a gradual increase in SA-β-gal expression was observed from 24 h to 14 days post-injury [23]. On the other hand, our results could explain those of Torres-Querol, who did not identify SA-β-gal in the brain at 3 days after tMCAO in mice [12]. These findings align with our own and support the idea that a reperfusion period longer than 3 days is necessary to detect the development of senescence-associated lysosomal dysfunction following brain focal ischemia.

The senescence-associated secretory phenotype, SASP, is another factor to consider. Our results show that three of the most studied cytokines of the SASP (IL-6, IL-1β, and TNF) are greatly overexpressed 24 h after I/R. However, this overexpression is not sustained at later time-points, from 3 to 14 days post-I/R. Our results are in accordance with those obtained by Torres-Querol, who did not find differences in mRNA expression of IL-6 and TNF at three days post-I/R in mice subjected to tMCAO [12]. Furthermore, in an in vitro model of neuronal replicative senescence, neither IL-1β nor TNF was detected in the culture media, and IL-6 was only minimally detected and exhibited no variation over time [24]. However, these authors identified an increase in other SASP factors, including Cxcl1, Cxcr2 [12], and MCP1 [24]. It has been extensively documented that the constituents of the SASP vary depending on the characteristics and duration of the stressor that triggers senescence, as well as the tissue in which senescence occurs [25,26,27]. It is therefore essential to gain a more precise understanding of the temporal expression of the components of the SASP in the brain following an ischemic stroke, rather than basing our studies exclusively on universally described cytokines, which are also associated with the earlier neuroinflammatory response to ischemic damage.

Alterations in both the structure and function of the nucleus are the main hallmarks of cellular senescence. Since such an issue has not been investigated in the context of ischemic stroke, we have addressed it by analyzing the expression of LB1, Chk1, and Chk2. The loss of LB1 has been proposed as a relevant molecular mechanism and marker in the development of cellular senescence [28,29,30]. Our results show a marked decrease in the total number of LB1-immunopositive cells within the infarct region. Unfortunately, we were unable to definitively determine which cell type(s) might be responsible for the overall senescence-associated reduction in the number of LB1-immunopositive cells. We also examined the morphology of LB1-labeled nuclear envelopes in the different cell types, revealing appreciable aberrantly shaped nuclei only in neurons of the peri-infarct region. Similar to our findings, other authors identified alterations in the morphology of Lamin A and C-labeled nuclei, indicative of a senescence-like phenotype, in the cortical neurons of aged rats [24]. In light of the aforementioned, it would be necessary to determine which components of the nuclear envelope are susceptible to alteration and the manner in which they are affected by the senescence process after brain ischemic stroke.

Another aspect concerning the nuclear dynamics of senescent cells is the cellular response to DNA damage (DDR). The senescent phenotype is also characterized by the presence of persistent nuclear foci containing an accumulation of DDR-specific factors, called DNA segments with chromatin alterations reinforcing senescence (DNA-SCARS). These DNA-SCARS are implicated in both senescence-mediated cell cycle arrest and SASP expression [31]. Proteins such as Chk1 and Chk2 can be part of these DNA-SCARS [32,33]. As far as we know, Chk1 and Chk2 expression in ischemic stroke has not been investigated. Our results show, with the exception of Chk1 in the caudate–putamen, an increase in the expression of Chk1 and Chk2 at 14 days post-I/R in the ischemic hemisphere, which may indicate that senescence-associated DNA-SCARS are produced following ischemic stroke.

One of the objectives of the present study was to ascertain the cellular types in which the senescent phenotype may be developing following an ischemic stroke. The heterogeneity of senescent markers across the cell types and tissues presents a challenge in making definitive categorical assertions. Focusing on neurons, there were significant increases in both the number and fluorescence intensity of p16-immunopositive cells. A recent study showed similar results in a tMCAO mouse model, wherein the authors observed increased p16 intensity in neurons at three days post-I/R [12]. These findings, together with our observation that neuronal nuclei show aberrant LB1 morphology, indicate that neurons undergo senescence associated with ischemic stroke. Of note, p16-positive neurons were also found in the non-ischemic hemisphere. It has been reported that some of the canonical cell cycle regulators of the Ink4 family, such as p16, are constitutively expressed in differentiated neurons of the neocortex [34] and hippocampus [35] of adult mice. In addition, p16 expression has been observed to increase during the in vitro terminal differentiation of NT2 cells into neurons [36]. The basal neuronal expression of p16 could play a role in establishing and maintaining the differentiated state of neurons, preventing them from re-entering the cell cycle. As for microglia/macrophages, we observed an increase in both the number and fluorescence intensity of p16-immunopositive cells, as well as an increase in the fluorescence intensity of p21 in the infarct region. These findings align with those of Torres-Querol, who found an elevation in both p16 and p21 intensity in microglia in a tMCAO mice model [12]. Furthermore, we observed that the accumulation of SA-β-gal is confined to the infarcted region, coinciding with the spatial localization of microglia/macrophages. The aforementioned, in conjunction with our previous observation that lipofuscin accumulation is also restricted to this region [11], suggests the possibility that microglia/macrophages also express a senescent phenotype associated with ischemic stroke. With regard to astrocytes, although we did not observe a significant number of astrocytes expressing p16 in the damaged brain 14 days after ischemic stroke in rats, previous studies have reported p16 expression in this cell type within the infarct area as soon as 24 to 48 h after ischemic stroke in mice [17]. In addition, a senescence phenotype (SA-β-gal) has been observed in cultured rat cortical astrocytes 24 h after oxygen–glucose deprivation/reoxygenation (OGD/R) [16]. Similarly, cultured mouse whole-brain astrocytes showed that p16 expression emerged 24 h after OGD/R [17]. This emphasizes the need for a comprehensive spatio-temporal characterization of brain cell senescence in ischemic stroke models. Interestingly, elevated levels of inflammatory factors (IL-6, TNF, and TLR-4) and premature cell senescence markers (p16 and p21) have been reported in distant organs such as bone marrow in mice after tMCAO, thus pointing toward remote cell senescence in addition to the local cerebral response [37].

Overall, our results lend support to the existence of brain cell senescence after ischemic stroke in neurons and microglia/macrophages. We provide evidence based on some biomarkers of cell cycle arrest, increased lysosomal activity, the senescence-associated secretory phenotype, and DNA/nuclear damage. However, there is still room to examine and identify more precisely the specific markers involved in the senescent phenotype after brain ischemic stroke. This would facilitate a deeper comprehension of the role of cellular senescence in the pathophysiology of ischemic stroke and, consequently, the implementation of senolytic therapy with greater guarantees of success. Senescent cells are in a state of irreversible cell cycle arrest and can resist apoptosis due to the upregulation of Bcl-2 family anti-apoptotic proteins. Senolytic drugs like navitoclax selectively induce apoptosis in senescent cells by inhibiting Bcl-2 family proteins, which could reduce the burden of senescent cells in brain tissue after ischemic stroke.

## 4. Materials and Methods

### 4.1. Animals and Ethical Issues

One hundred and five male 12-week-old Wistar rats from Charles River Laboratories (Barcelona, Spain), housed under standard conditions with food and water ad libitum, were used in this study. All animal experiments complied with ARRIVE (Animal Research: Reporting of In Vivo Experiments) guidelines. Experiments were conducted in compliance with the legislation on the protection of animals used for scientific purposes in Spain (RD 53/2013) and the EU (Directive 2010/63/EU). Protocols were approved by the Ethics Committee on Animal Experimentation from Instituto de Investigación Sanitaria La Fe.

### 4.2. The tMCAO Model: Focal Cerebral Ischemia–Reperfusion

Anesthesia was induced (5 mg/kg diazepam, 100 mg/kg ketamine, and 0.3 mg/kg atropine, i.p.) and maintained by a facemask with 0.5–1% sevoflurane in 80% medical air plus 20% O_2_. tMCAO (60 min) was performed by following the intraluminal suture procedure [38] adapted to our experimental setup [39]. During the procedure, cortical perfusion (CP) was continuously monitored to confirm the presence of a correct pattern of ischemia–reperfusion. CP was measured by means of a laser-Doppler blood flow probe (Perimed System, Stockholm, Sweden) positioned in the parietal cortex (2 mm caudal, 3.5 mm right lateral from bregma). To confirm that all animals were similarly affected, a neurofunctional assessment was performed at 24 h of reperfusion (Supplemental Material). At 24 h and 3, 7, and 14 days post I/R, the animals were euthanized under deep anesthesia to obtain the brain and process it according to specific requirements for each determination (Supplemental Material).

### 4.3. Exclusion Criteria and Experimental Groups

Sixty-three rats were excluded from the study as they fulfilled one of the following exclusion criteria: (1) no ischemia (CP reduction < 50% from baseline, *n* = 2 [1.9%]); (2) no reperfusion (CP did not reach the baseline after filament withdrawal, *n* = 14 [13.3%]); (3) death before the time limit established for each experimental group (*n* = 33 [31.4%]); and (4) no infarction in spite of a right I/R pattern (*n* = 14 [13.3%]). Therefore, the following experimental groups were established depending on the reperfusion time: 24 h post-I/R (*n* = 9), 3 days post-I/R (*n* = 9), 7 days post-I/R (*n* = 10), and 14 days post-I/R (*n* = 14).

### 4.4. SA-β-Gal Staining and Quantification

Fresh frozen cryostat-cut brain coronal sections 0.2 to −1.8 mm from the bregma were obtained (20 μm thick; CM 1950 cryostat, Leyca Biosystems, Nussloch, Germany). SA-β-gal staining was carried out by using the Senescence β-Galactosidase Staining Kit (#9860, Cell Signaling, Danvers, MA, USA) according to the manufacturer’s instructions. Briefly, sections were fixed and then incubated with the β-Galactosidase Staining Solution (pH = 6; overnight at 37 °C). Afterward, sections were counterstained for 10 min with 0.1% Nuclear Fast Red (NFR, Sigma-Aldrich, Darmstadt, Germany). ImageJ NIH software version 1.53t was used for quantification by an investigator blinded to the selected regions.

### 4.5. Immunofluorescence Staining

At the end of the corresponding reperfusion time, animals were anesthetized with 5 mg/kg diazepam and 100 mg/kg ketamine and perfused with a cold saline solution for 10 min, followed by 20 min of 4% paraformaldehyde (PFA). Thereafter, brains were collected, post-fixed for 24 h in 4% PFA, washed with cold PBS, and cryoprotected in 30% sucrose. Cryoprotected brains were flash-frozen on dry ice, mounted in Tissue-Tek OCT compound (Sakura Finetek, Torrance, CA, USA), cryostat-cut into 20 µm thick coronal sections (0.2 to −1.8 mm from bregma), and stored at −20 °C.

Selected slices were incubated with sodium borohydride for 30 min, blocked with 10% normal goat serum (#S-1000-20, Vector Laboratories, Newark, CA, USA) in PBS with 0.8% Triton X-100 for 1 h at room temperature, and then incubated overnight at 4 °C with the following primary antibodies: (1) anti-p16 ([1:250], sc-1661, Santa Cruz Biotechnology, Dallas, TX, USA); (2) anti-p21 ([1:250], 28248-1-AP, Proteintech, Rosemont, IL, USA); (3) anti-LB1 ([1:100], sc-374015, Santa Cruz Biotechnology, Dallas, TX, USA); (4) anti-NeuN ([1:250], mAb #12943, Cell Signaling, Danvers, MA, USA); (5) anti-GFAP ([1:500], #840001, Biolegend, San Diego, CA, USA); and (6) anti-Iba1 ([1:500], #019-19741, Fujifilm Wako Pure Chemical Corporation, Chuo-ku, Osaka, Japan). Afterward, the sections were incubated for 1 h at room temperature with the corresponding secondary antibodies: (1) Alexa Fluor 594 goat anti-mouse IgG ([1:200], #A11005, Invitrogen, Waltham, MA, USA); (2) goat anti-rabbit IgG, DyLight 488 ([1:200], # DI-1488-1.5, Vector Laboratories, Newark, CA, USA); or (3) Alexa Fluor 568 goat anti-rabbit IgG ([1:200], #A11036, Invitrogen, Waltham, MA, USA), followed by nuclear staining with DAPI (#A1001, ITW Reagents, Milan, Italy) and mounting with FluorSave (#345789, Millipore, Darmstadt, Germany). For the immunostaining of p21, slices were first subjected to antigen retrieval with citrate buffer 10 mM, pH 6 (2 cycles of 6 min at 85 °C, followed by 10 min on ice).

For quantification, two regions were selected in the ipsilateral hemisphere of the brain: the infarct zone and the peri-infarct zone. The contralateral hemisphere was used as a control. Three images (40×, 36,457.5 µm^2^) were taken from each region using a high-resolution confocal microscope LSM 980 (ZEISS, Oberkochen, Germany). The number of immunopositive cells was expressed as a percentage of the corresponding DAPI-positive cells or the corresponding NeuN-, Iba1-, or GFAP-positive cells. For fluorescence intensity measurement, three cells were randomly selected from each image. Quantification was performed by an investigator blinded to the selected regions, using ImageJ NIH software version 1.53t for cell counting and ZEISS ZEN 3.9 software for fluorescence intensity measurement.

### 4.6. RT-qPCR Analysis of Gene Expression

A 2 mm thick brain coronal section (0.2 to −1.8 mm from bregma) was obtained, and both ischemic and non-ischemic cortex and caudate–putamen samples were dissected, flash-frozen in liquid N_2_, lyophilized, and ground to obtain brain powder. Total RNA was isolated using the TRIZOL reagent according to the manufacturer’s instructions (Sigma-Aldrich, Darmstadt, Germany). The cDNA used as a template for amplification in the qPCR assay was obtained by the reverse transcription reaction using the PrimeScriptTM RT reagent Kit (Takara Bio, Kusatsu, Japan) according to the manufacturer’s protocol. The gene expression was analyzed using TB Green^®^ Premix Ex TaqTM or Premix Ex TaqTM kits for primers or taqman probes, respectively (Takara Bio, Kusatsu, Japan) in a ViiA 7 Real-Time PCR System (Thermo Fisher Scientific, Waltham, MA, USA). Each reaction was run in triplicate, the threshold cycle was determined, and the relative gene expression was calculated with the Schmittgen and Livak comparative method [40], using *Actb* as a reference gene. The specific primers and Taqman probes used are shown in Table 1.

### 4.7. Statistical Analysis

Data are expressed as mean ± standard error of the mean (SEM). Data analysis was performed using GraphPad Prism 8 software (GraphPad Software, Boston, MA, USA). Statistical comparisons were assessed by (1) one-way analysis of variance (ANOVA) followed by Dunnet’s post hoc test (three-region immunofluorescence), (2) two-tailed Student’s *t*-test (two-region immunofluorescence), and (3) two-way ANOVA followed by Tukey’s post hoc test (SA-β-gal expression) or Sidák multiple comparison test (mRNA expression). Differences were considered significant at *p* < 0.05.

## Figures and Tables

**Table 1:** Taqman probes and primers used for RT-qPCR.

Target Gene	Taqman Probe	Forward Primer Sequence (5′–3′)	Reverse Primer Sequence (5′–3′)
*Chk1*		CATGTTTCCAGTTGGCCTCT	TCTTCTTGTCTGGGCGACTT
*Chk2*		CTTTCGCATCTTCAGGGAAA	AGTGAAAGTGCGATTTCAGAGTT
*Actb*		TTCAACACCCCAGCCATGT	GTGGTACGACCAGAGGCATACA
*Cdkn2a*/*p16*	Rn00580664_m1		
*Cdkn1a*/*p21*	Rn00589996_m1		
*Tp53*	Rn00755717_m1		
*Il6*	Rn01410330_m1		
*Il1b*	Rn00580432_m1		
*Tnfa*	Rn01525859_g1		
*Actb*	Rn00667869_m1		

## Data Availability

The raw data supporting the conclusions of this article will be made available by the authors on request.

## Supplementary Materials

The following supporting information can be downloaded at https://www.mdpi.com/article/10.3390/ijms26052364/s1.

## Abbreviations

The following abbreviations are used in this manuscript:

Chk1	Checkpoint kinase 1
Chk2	Checkpoint kinase 2
CP	Cortical perfusion
DDR	DNA damage response
DNA-SCARS	DNA segments with chromatin alterations reinforcing senescence
I/R	Ischemia–reperfusion
LB1	Lamin B1
NFR	Nuclear fast red
PFA	Paraformaldehyde
SA-β-gal	Senescence-associated β-galactosidase
SASP	Senescence-associated secretory phenotype
tMCAO	Transient middle cerebral artery occlusion
TTC	2,3,5-Triphenyltetrazolium chloride
