# Supplementary material for: Spatio-Temporal Characterization of Cellular Senescence Hallmarks in Experimental Ischemic Stroke
Source: Int J Mol Sci. 2025 Mar 6;26(5):2364. doi: 10.3390/ijms26052364 (PMC11900039; doi:10.3390/ijms26052364)
Supplement: Supplementary file 1 [file ijms-26-02364-s001.zip › ijms-3488005-supplementary.pdf]

Supplemental Material

Assessment of ischemic brain damage: Neurofunctional score and brain infarction

The severity of functional impairment was evaluated at 24 hours post-reperfusion through a comprehensive assessment of: (a) spontaneous activity; (b) circling to the left; (c) parachute reflex: protective abduction of forelimbs; and (d) resistance to left forepaw stretching. The total score may vary from 0, indicating the absence of neurological deficits, to 5, which represents the most severe neurological deficits.

To confirm the existence of brain infarction, animals were euthanized under anesthesia by intracardiac injection of KCl (200 mg/kg) or perfusion with paraformaldehyde (PFA, 4%), and the whole brain was removed. According to the specific requirements, two histological techniques were followed: 1) fresh coronal sections (2 mm-thick) were stained with 2,3,5-triphenyltetrazolium chloride (TTC; Sigma-Aldrich, Darmstadt, Germany); and 2) frozen coronal sections (20 µm-thick) were stained with thionin solution (ChemCruz, Dallas, TX, USA).

Table S1 Physiological parameters, cortical perfusion (CP), and neurological score

	24 h post I/R			3 days post I/R		
**Parameter**	**Baseline**	**Ischemia**	**Reperfusion**	**Baseline**	**Ischemia**	**Reperfusion**
**CP (%)**	100	44.2 ± 21.5	130.4 ± 60.6	100	34.8 ± 11.4	104.7 ± 50.6
**MABP (mmHg)**	127.1 ± 11.5	131.2 ± 17.3	117.9 ± 31.5	116.6 ± 18.3	124.5 ± 8.3	120.2 ± 11.6
**Glucose (mg/dL)**	132 ± 13.9	154.3 ± 20.8	153.5 ± 20.6	126.7 ± 0.6	157 ± 19.5	155.5 ± 12
**Temperature (ºC)**	35.9 ± 0.4	36.4 ± 0.9	37.4 ± 1.7	35.7 ± 0.2	35.7 ± 0.1	36.7 ± 1.5
**Weight (g)**		340.6 ± 17.8			338.4 ±21.8	
**Neurological score (24 h post I/R)**		3 [2,4]			3 [2,4]	

	7 days post I/R			14 days post I/R		
**Parameter**	**Baseline**	**Ischemia**	**Reperfusion**	**Baseline**	**Ischemia**	**Reperfusion**
**CP (%)**	100	38.1 ± 15.2	130.9 ± 54.7	100	32.2 ± 22.7	117.4 ± 40.7
**MABP (mmHg)**	147.5 ± 14.9	138.4 ± 8.4	131.9 ± 15.9	120.1 ± 13.1	119.3 ± 9	101.9 ± 19.1
**Glucose (mg/dL)**	137.6 ± 15.5	151.8 ± 4.4	135.6 ± 15.4	150.3 ± 39.1	156.2±55	163 ± 78.9
**Temperature (ºC)**	36.3 ± 0.5	35.6 ± 1.4	37.4 ± 1.3	35.9 ± 1.7	36.7 ± 1.2	37.1 ± 1.4
**Weight (g)**		359.3 ± 34.5			349.4 ± 72.5	
**Neurological score (24 h post I/R)**		3 [2,5]			3 [1,5]	

Physiological parameters and cortical perfusion (CP) measurements during the three stages of transient middle cerebral artery occlusion (tMCAO: baseline, ischemia and reperfusion) in the four experimental groups. Weight was measured before tMCAO and neurological score was assessed 24 hours post I/R in all animals included. Data are expressed as mean ± SEM, except for neurological score which is expressed as median [Q1, Q3], of *n* = 9 (24 h post I/R), *n* = 9 (3 days post I/R), *n* = 10 (7 days post I/R) and *n* = 14 (14 days post I/R). I/R, ischemia/reperfusion; MABP, mean arterial blood pressure.

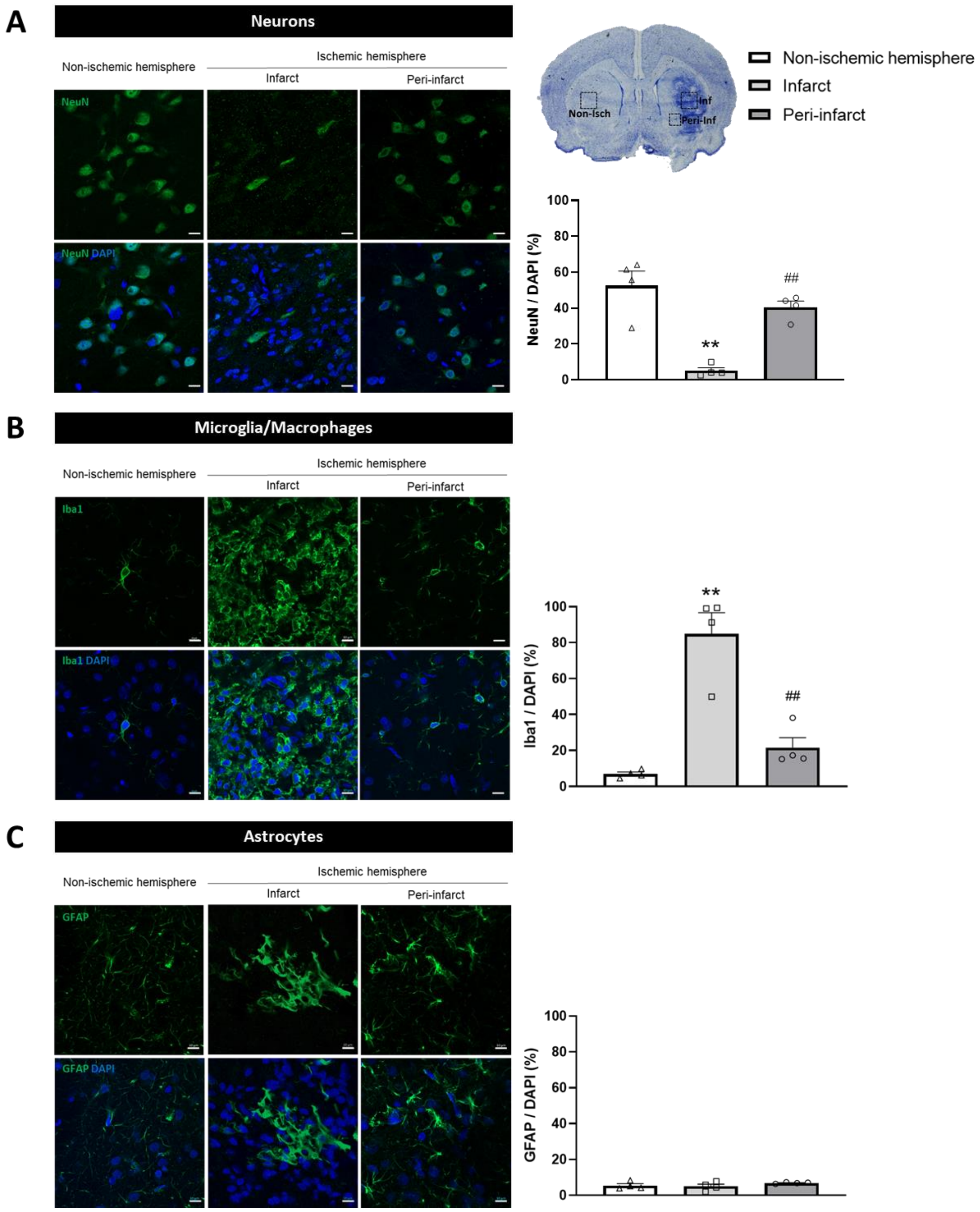

Figure S1. Distribution of the main neural cell types after ischemic stroke in the non-ischemic (triangles) and ischemic hemisphere (infarct [squares] and peri-infarct [circles] regions) at 14 days post I/R. Immunofluorescent representative images (left) and quantification (right) of (**A**) neurons (NeuN), (**B**) microglia/macrophages (Iba1) and (**C**) astrocytes (GFAP) in the three studied regions. Data are expressed as the mean ± SEM from n = 4 animals. Scale bar = 10 µm. Statistical differences were determined by one-way ANOVA followed by Tukey's multiple-comparisons test; $^{**}p < 0.01$ significantly different from the non-ischemic hemisphere; $^{\#\#}p < 0.01$ significantly different from the infarct region. I/R, ischemia/reperfusion.
